# Adaptation required to preserve future high-end river flood risk at present levels

**DOI:** 10.1126/sciadv.aao1914

**Published:** 2018-01-10

**Authors:** Sven N. Willner, Anders Levermann, Fang Zhao, Katja Frieler

**Affiliations:** 1 Potsdam Institute for Climate Impact Research, Potsdam, Germany.; 2 Institute of Physics, Potsdam University, Potsdam, Germany.; 3 Columbia University, New York, NY 10964–8000, USA.

## Abstract

Earth’s surface temperature will continue to rise for another 20 to 30 years even with the strongest carbon emission reduction currently considered. The associated changes in rainfall patterns can result in an increased flood risk worldwide. We compute the required increase in flood protection to keep high-end fluvial flood risk at present levels. The analysis is carried out worldwide for subnational administrative units. Most of the United States, Central Europe, and Northeast and West Africa, as well as large parts of India and Indonesia, require the strongest adaptation effort. More than half of the United States needs to at least double their protection within the next two decades. Thus, the need for adaptation to increased river flood is a global problem affecting industrialized regions as much as developing countries.

## Teaser

United States and Europe must adapt to climate-induced river flood risk at same level as India, Indonesia, and parts of Africa.

## INTRODUCTION

Fluvial floods are among the most common and devastating natural disasters worldwide (*1*). Flood events were the main cause of internal displacement in 2008 to 2015 (*2*), affecting people’s assets and physical well-being, as well as regional infrastructure and economy. Globally, the physical hazard (for example, flood volume) (*3*) and exposure to floods in terms of people and properties (*4*) have increased. Assuming fixed population, people affected by 100-year return period floods are projected to increase in a warmer future (*5*). At the same time, global fatalities and direct damages from floods have stabilized or decreased since 1990, reflecting success in flood protection measures (*3*). However, on a global scale, a comprehensive assessment of the regional adaptation requirements was lacking the incorporation of spatially heterogeneous flood protection: Generally, studies either assume no protection (*3*) or assume a universal flood return period (*5*). By contrast, real flood protection varies greatly across different regions and countries for various reasons (for example, economic ability and political will toward protection investment). In light of possible future flood events, a number of countries with a large population and strong economic performance (for example, India and the United States) require extensive adaptation measures to keep flood risk at its present level.

Here, we compute the increase in flood protection that is required to keep the observed high-end flood risk of the past constant in the next 25 years. We relate these changes to the existing protection level (*6*) that was chosen by the different societies in the different regions in their specific assessment of vulnerability and risk. Exposure is calculated based on a fixed present-day distribution of population. Changes in risk are derived from changes in flood hazards computed from a multimodel ensemble of climate models and hydrological models within the Inter-Sectoral Impact Model Intercomparison Project (ISIMIP) framework (*7*). The inertia in the climate system makes it possible to predict, within model uncertainty, changes in flood hazards up to the year 2040, independent of the specific carbon emission pathway that is chosen by society within the next 25 years (*8*).

### Method summary

**Fig. 1:**
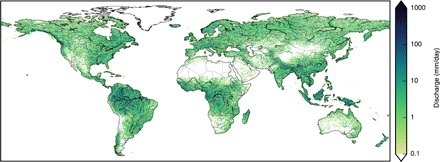
Mean daily discharge (historic period, 1971 to 2004). Median over the ensemble of all 50 combinations of 10 hydrological and 5 climate models. Their runoff output was routed by the river routing model CaMa-Flood (*12*) to derive their mean daily discharge in the historic period; plotted here is the median of all model combinations. Very dry cells (discharge, <0.1 mm/day) are masked (white); this mask is also used for the other figures.

Our computation is based on a multimodel ensemble of all 50 naturalized runs of 10 hydrological models (GHMs) and bias-corrected (*9*) daily forcing from five different global climate models (GCMs) (*10*) under historical emissions and the four representative concentration pathways (RCPs) (*11*). Using the river routing model CaMa-Flood (*12*), we estimate for each model combination’s runoff time series the annual maximum daily flood discharge in two periods: a historic period, 1971 to 2004, and a future period, 2035 to 2044 (the ensemble median of mean daily discharge is shown in Fig. 1). We then fit an extreme value distribution to the historic time series of each model run (34 data points). Following a novel approach (see Materials and Methods), we subsequently correct for natural variability and selection bias in the historic period by incorporating 12 realizations in a preindustrial control run of 439 years total length. This method yields an ensemble of extreme value fits to estimate the return period value of each historical and future data point. To correct for model-specific bias, we translate the resulting return period into flood depth using a historical simulation forced by observed climate variables (*5*). The flood depth is then downscaled to yield a flooded area at 2.5′ resolution and multiplied by spatially explicit population data to yield the number of affected people. The population distribution is kept constant based on the 2010 values (*13*). Regionally specific present-day protection measures are accounted for by considering only flood events where discharge exceeds the spatially explicit protection level from the FLOPROS database (*6*). This combines empirical data about existing flood protection infrastructure and protection standards and requirements set by policy with modeled data to achieve detailed, global coverage. For each of the two temporal periods and each subnational region, a distribution of the number of affected people is obtained from the different model simulations corrected for each realization. We define the high-end flood risk as the 90th percentile of that distribution, that is, the absolute number of people affected by the 90th percentile of the flood events in the corresponding period. Finally, the additional protection that is required to keep the historic high-end flood risk in the future period is interpreted as the adaptation required. This is to be interpreted in the sense of flood protection level in the FLOPROS database, that is, a mixture of physical protection and policy requirements. We show the median of all 12 realizations (correction periods drawn from the preindustrial control run) and the 16.7th and 83.3rd percentile as the lower and upper bound of the “likely” range as defined by the Intergovernmental Panel on Climate Change.

## RESULTS

**Fig. 2:**
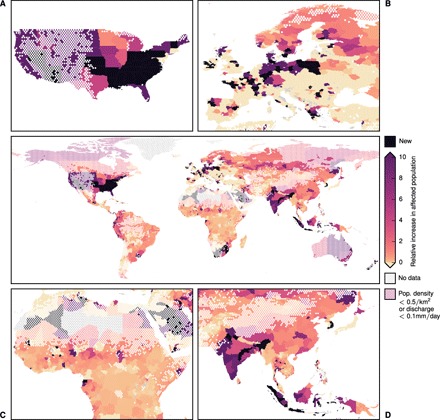
Relative increase in affected people without adaptation measure (realization ensemble median). Increase is given as the multiple of change between future and historic periods relative to the historic period. A value of 2 means that three times as many people are at risk of high-end river flooding during 2035 to 2044 compared to 1971 to 2004. Regions with affected population in the future period, but none in the historic one, are marked “new” (black). Subfigures show regional foci on the (**A**) United States, (**B**) Europe, (**C**) Africa, and (**D**) Southeast and East Asia. Decrease in affected population is cut off to 0. Pop. density, population density.

**Fig. 3:**
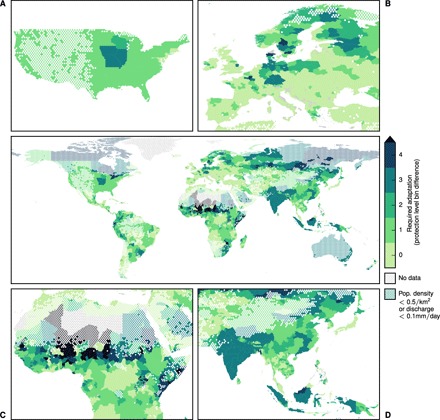
Increase in the regional flood protection level required to preserve the current high-end flood risk for the period 2035 to 2044 (realization ensemble median). Additional protection is given in levels, starting with 0 for regions without adaptation need. Level boundaries are 0, 1, 2, 4, 8, 16, 32, 64, 128, 256, 512, and 1000 years return period. Numbers shown are absolute difference in level numbers to current protection per subnational region in the FLOPROS database (*6*). Subfigures show regional foci on the (**A**) United States, (**B**) Europe, (**C**) Africa, and (**D**) Southeast and East Asia.

During the next 25 years, the high-end flood risk will strongly increase in all equatorial regions, Northern America, Northern Europe, and Northeast Asia (Fig. 2). Because of the associated uncertainty, especially arising from the extreme value distribution fit, we sort current and additional protection measures into levels bounded by powers of 2 in years return period (0, 1, 2, 4, 8, 16, 32, 64, 128, 256, and 512; lower bound included); numbers given are median and the minimum and maximum 33rd percentile likely range around it in brackets [16.7th percentile; 83.3rd percentile] (see Materials and Methods for details). The associated increase in protection that is required to keep the additionally affected population below zero is given in number of protection levels in Fig. 3 (corresponding lower and upper bounds of the likely range are shown in figs. S1 and S2). As an example, for a region protected against 20-year floods, an increase by 2 protection levels means a necessary protection against at least 64- to 128-year events. Depending on current protection levels, this number can thus have very different qualitative meanings for different regions. We also show relative increase in protection (fig. S3), number of affected people in the historic (fig. S4) and in the future period (fig. S5), and absolute increase in the affected population (fig. S6). In the following, we present our results for an exemplary choice of regions per continent and conclude with a generalization as far as this is possible here. Here, we use the word “affected” in two different ways, each referring to the future period unless stated otherwise. First, the affected population of a region is the absolute number of people at the 90th percentile of all people experiencing flood events in a period, that is, people living in areas flooded by particular events. Second, an affected region is a region with affected population. As explained in Discussion, these numbers are subject to a set of assumptions, especially that of changing physical flood exposure while keeping human factors constant.

For the United States (Figs. 2A and 3A), 42 of the 50 states, and the District of Columbia, will experience an increased flood risk if no additional protection measures are taken. To keep their high-risk protection, a number of states with a 100-year protection level will have to increase their protection: Illinois, Iowa, and Missouri by 3 [3; 3] levels; Wisconsin by 2 [1; 3] levels; and Minnesota by 1 [1; 2] level. Unlike smaller East Coast states, 30 states with 500-year protection need to increase protection by 1 additional level. In Europe (Figs. 2B and 3B), the strongest adaptation need arises in a band near the Baltic Sea including Sweden, Germany, and Poland. Germany in total needs to adapt to an increase in flood risk with 710,000 people affected in the future period [320,000; 1.6 million, to be denoted “M” in the following] compared to 76,000 [20,000; 320,000] in the historical period, especially in north Germany. In Poland, the northern Voivodeships’ current protection is not exceeded in the historical period. To keep the high-end flood risk at zero, German and Polish coastal regions have to increase the protection by a range between 1 and 3 levels. In France, the areas along Rhone, Loire, and Seine—total risk increase 8000 [4000; 59,000] to 128,000 [12,000; 540,000] people—need to increase their protection by at least 1 level.

In Africa (Figs. 2C and 3C), adaptation needs to be increased from the generally low protection level currently in place. The largest adaptation need will arise in the Niger River basin (inland and coastal deltas), Nile, and Zambezi. For Nigeria, we observe an almost 50% increase (4M [2M; 5M] to 6M [5M; 7M]) of high-end flood risk. In Mali (1.1M [1.0M; 1.2M] to 1.5M [1.4M; 1.6M]), regions along the Niger River would need more than 2 levels of adaptation, given the current low protection of 2 to 3 years (excluding Bamako). In Chad, a total rise in affected people (0.7M [0.6M; 1.2M] to 1.4M [1.2M; 1.8M]), particularly in the southwestern regions, is to be expected. Egypt shows the highest increase in people under high-end flood risk globally on a country level (0.2M [0; 1.1M] to 3M [0.9M; 8.3M]) with an adaptation need of 1 to 3 levels. Governorates along the Nile are affected, most of which will experience a flood risk beyond 100,000 people for the first time (for example, Suhaj, 0.2M [0; 0.6M]). Cairo (in Al Qahirah region) itself is sufficiently protected with a 100-year protection. Further upstream of the Nile, Sudan (0.7M [0.5M; 1M] to 1.3M [1M; 1.4M]; 2 to 6 levels of protection increase needed for keeping risk at the present-day level), South Sudan (1.2M [1M; 1.3M] to 1.4M [1.3M; 1.5M]; 1 to 3 levels of protection increase needed), and Uganda (1.4M [1.3M; 1.5M] to 1.6M [1.5M; 1.7M]) will be most affected in the future.

Asia (Figs. 2D and 3D) is the continent with the largest historical high-end flood risk as well as the strongest increase in that risk. Historically, Pakistan is already highly affected and will observe almost a doubling in high-end flood risk on a subnational level, from 6M [4M; 8M] to 11M [9M; 13M] people without additional protection. All of its eight provinces are already affected in the historic period. Five of them show an increase of more than 10% within the next 25 years. Most affected are the eastern provinces, Punjab (4.6M [3.2M; 6.4M] to 9.5M [7.7M; 11.2M]) and Sind (0.8M [0.8M; 1.2M] to 1.3M [1.2M; 1.7M]), which need an increase in protection by 3 [2; 3] and 2 [2; 3] levels, respectively. A similarly strong flood risk and adaptation need can be seen in 26 of India’s 36 states and union territories, 3 of which exceed 1M people. Most affected is the province of Bihar (1.8M [0.6M; 5M] to 9.2M [5.4M; 18.7M]). Most of the affected states show an adaptation need of 3 additional protection levels.

China will observe an increased high-end flood risk from 24M [18M; 34M] to 55M [46M; 69M] affected people. All but Shanghai show an increase in high-end risk of at least 20%; 14 of 31 provinces will have more than a million people each under high-end risk. Even the comparably strongly protected Hubei (100-year protection as opposed to the average of 20 years over the whole of China) will face a 10-fold increase (from 0.3M [0.1M; 1M] to 3.3M [2M; 4M] people) if the protection is not increased by 3 [3; 3] levels. The most affected regions are close to Shanghai, which itself is not affected by river flood (currently protected against 200-year floods). Guangdong, the province surrounding Hong Kong, is furthermore heavily affected and shows a massive increase from 0.7M [0.7M; 1.4M] to 3.8M [3.2M; 5.1M] people, with an adaptation need of 3 [2; 4] levels. Hong Kong itself is not expected to show an increase in high-end risk.

Almost constant flood risk is expected in Cambodia. Half the affected population lives in the province of Kândal with 2M [2M; 2M] people under high-end risk, directly surrounding Phnom Penh (currently protected against 24-year floods), itself not affected in neither the historic nor the future period. Vietnam is strongly affected, historically and in the future (10M [4.7M; 18.2M] to 17.9M [14.5M; 21.5M]), especially the Đông Nam Bộ region (2.7M [0.3M; 2.7M] to 2.8M [2.7M; 2.9M], 2 [1; 2] levels of adaptation needed) and Hồ Chí Minh City (0.2M [0.2M; 2.7M] to 2.7M [2.7M; 2.7M]). Here, the required adaptation is smaller than 1 level (0 [0; 1] levels), which is due to the already high risk in the historical period and only a very slight trend in time. Overall, 53 of 63 provinces are under increased high-end risk. The main island Java is the most affected region in Indonesia, although its current protection against 100-year events is comparably high. Eastern Java, Jawa Timur, shows the largest increase from 0 [0; 36,000] to 300,000 [199,000; 705,000] people affected with strong adaptation need (3 [2; 3] levels).

In addition, Central and South America show increased flood risk. For Mexico, we find an almost 25% increase in flood risk (450,000 [120,000; 870,000] to 560,000 [300,000; 960,000] affected people), with the strongest adaptation need around Mexico City, whereas the capital itself is under no additional risk. A similar difference between metropolitan and surrounding areas can be observed for Argentina’s capital, Buenos Aires: The Buenos Aires region needs 1 [1; 1] additional protection level to keep its current value of 99,000 [79,000; 461,000] people under high-end risk. However, Buenos Aires City is already sufficiently protected. The country’s overall increase in affected people is more than a doubling from 0.6M [0.3M; 1.2M] to 1.3M [0.8M; 1.8M]. In Brazil, states in the Paraná River basin are most affected. This especially includes the highly populated São Paulo state, for which we expect a 55% increase in people under high-end flood risk (0.3M [0.1M; 0.6M] to 0.5M [0.3M; 0.8M]) without adaptation. An increase by 1 [0; 1] protection level would keep the future risk at its present level.

In summary, we find pressure for adaptation to high-end flood risk widely distributed around the globe. We identify two qualitatively different challenges. On the one hand, regions with an already high level of flood protection have a low flood risk but need to adapt their protection to keep that risk low in the future. On the other hand, many regions with low protection level are already at high flood risk, which will, in many cases, increase in the future. We find this distinction not only between industrial and developing countries but also for cities and their rural surroundings.

## DISCUSSION

It should be noted that our investigation is an estimate based on the current state of precision that can be obtained on a global level. The exact numbers in the results should be interpreted with caution because of several methodological challenges. On the one hand, GHMs do not resolve a number of small-scale processes (*14*). On the other hand, GCMs have uncertainty in the representation of extreme precipitation events because of their spatial scale. To account for this, we combine climate models that are bias-corrected toward an observation-based data set (*15*) using a trend-preserving method (*9*) with a number of different hydrological models. Another caveat that the present study shares with previous studies is that the extreme value statistics is limited by a short time period of 34 years. We address this uncertainty by correcting the short time periods to a much longer preindustrial control run (a more detailed description of the uncertainty analysis for which we developed a new method is provided in the Supplementary Materials). In a few regions, the 90th percentile is dominated by few climate models (figs. S7 to S12). Given that we look at possible high-end risk, we chose to include those outcomes nevertheless in our statistics for these regions.

Our computation of the increase in high-end flood risk is fully consistent with previous results (*5*). Nevertheless, by using return periods (in terms of the historical period) as a measure for protection, one only accounts for an increase in flood intensity, not in frequency or duration of flood events during a year. Therefore, the actual increase in affected people may be even higher. Moreover, in particular, cities are often susceptible to flash floods, which may compound river floods, causing a higher risk. These are not captured within our analysis. An additional compounding impact is further possible from storm surge, a common issue for coastal cities. Therefore, a consideration of river flood risk alone is likely to underestimate the adaptation pressure for cities especially at the coast.

The simulations we analyzed were conducted without adaptation; we only incorporated the first-order response through the application of flood protection. We further assume that the risk aversion in the regions remains the same in the next 25 years. If that risk aversion increases due to socioeconomic developments, then higher protection might be necessary. The same holds for other factors affecting decisions on protection levels such as financial restrictions, as well as changes in population size and distribution, which are held constant in this study. In addition, economic development is a very important driver of flood risk changes, especially in urban areas (*16*). Although we here study adaptation in terms of protection level (physical as well as policy requirements), other adaptation measures exist, such as insurance schemes and guided economic and structural development.

Here, we show that the adaptation need is similarly high in highly developed countries in North America, Europe, and East Asia, as well as for developing countries in Africa, Asia, and South America. Many areas around big cities are strongly affected; these include the regions around Shanghai, Hong Kong, Jakarta, Mexico City, São Paulo, Buenos Aires, and Cairo (zoomed-in views of these regions that stand out in this regard are shown in fig. S13). To keep the high-end flood risk at a level to which the regional population is accustomed, strong adaptation measures are often required in regions all around the globe. In any case, to avoid unmanageable climate damages in the second half of this century, it is most likely necessary to keep in line with the Paris climate agreement (*17*).

## MATERIALS AND METHODS

### Climate data

In the framework of the ISIMIP (*7*), a set of five different GCMs (HadGEM2-ES, IPSL-CM5A-LR, MIROC-ESM-CHEM, GFDL-ESM2M, and NorESM1-M) participating in the CMIP5 project (*10*), at daily 0.5° resolution, were bias-corrected toward an observation-based data set (*15*) using a trend-preserving method (*9*) that facilitates climate change studies. These models’ output was used as the climatic forcing.

### Flood model

A set of eight GHMs, one global land surface model, and one dynamic global vegetation model (summarized as GHMs) were driven by the climate forcing mentioned above to generate projections of future floods. Specifically, we used global 0.5° gridded daily runoff results from the DBH (*18*), H08 (*19*), Mac-PDM.09 (*20*), MATSIRO (*21*), MPI-HM (*22*), PCR-GLOBWB (*23*), VIC (*24*), and WBMplus (*25*) hydrological models; the JULES (*26*) land surface model; and the LPJmL (*27*) dynamic global vegetation model (see table S1 for further model details). GHMs were run without direct coupling to GCMs; thus, potential feedbacks were not represented. The simulations used (from the “nosoc” experiment, no socioeconomic changes) did not consider human interference, which likely affects drought more than floods (*28*). Further details about the GHM simulations can be found in the ISIMIP simulation protocol available at www.isimip.org.

For 34 combinations, we used RCP2.6, RCP4.5, RCP6.0, and RCP8.5 as future projections, whereas for 16 combinations (GCMs GFDL-ESM2M, IPSL-CM5A-LR, MIROC-ESM-CHEM, and NorESM1-M in combination with the GHMs DBH, Mac-PDM.09, MATSIRO, and VIC), only RCP2.6 and RCP8.5 were available.

To harmonize the output of the different hydrological models with respect to their river network, we used the river routing model CaMa-Flood (version 3.44) (*12*). We also included CaMa-Flood because it agrees better with observed river discharges, especially for peak values, than the direct output of the hydrological models (*28*). This was driven by each model combination of daily runoff data (50 combinations each for historical and each available future projection) to derive daily discharge at 0.25° global grids. The first year of historical (1971) and future projection (2006) runoff was repeated five times as spin-up for CaMa-Flood. We then derived the annual maximum daily discharge for each grid cell.

### Flood return periods

For each simulation and each grid cell, we fitted the generalized extreme value (GEV) distribution (*29*) to the historical time series of the annual maximum daily discharge using L-moment estimators (*30*) of the distribution parameters; for small sample sizes like here (34 data points; 1 for each year), these show substantially smaller SDs than maximum likelihood estimators (*31*, *32*), which were also computed for comparison. Cells with a mean daily discharge of less than 0.1 mm/day of the historical model period were excluded, because they have insufficient data density and are not important for flood studies.

The analysis was limited by the number of available data for the fitting of the extreme value statistics. We developed a method to correct for natural variability and bias in the selection of the small historical sample; a schematic overview for the computational chain from discharge via return period to number of affected people is shown in fig. S14; a detailed description is given in the following paragraphs.

For the correction, we used a preindustrial control run (“picontrol”) of the climate model GFDL-ESM2M in combination with the hydrological model LPJmL (note that simulations were only available for this model combination). This simulation yielded 439 years (1661 to 2099) of discharge output from CaMa-Flood, which follows the GEV distribution very well in nondry areas; fig. S15 shows the probability plot correlation coefficient (*33*) of the corresponding GEV fit per grid cell, where we used unbiased plotting positions (*i* − α)/(*N* + 1 − 2α) with α = 0.4 as recommended for general distributions (*34*).

We further separated the long preindustrial control run into 12 disjoint realizations of 34 consecutive years each (1661 to 1694, 1695 to 1728, etc.); fig. S16 shows the set of distributions at four representative grid cells. For each of these realizations, we computed the adaptation pressure as if the realization represented the historic time series corrected to the much better fit to the overall 439-year run. This yielded an ensemble of 12 realizations, which we used to derive the median and the uncertainty due to extreme value fitting to only 34 data points in the end results. To our knowledge, such an analysis incorporating uncertainty has not been done yet in this field because of lack of time series of sufficient lengths.

The correction was done assuming that between models, only return period can be transferred; other variables such as discharge are model-specific. For a return period in GCM,GHM, we derived the corresponding return level discharge in realization *r* in GFDL-ESM2M,LPJmL; the return period used in the bias correction was then derived using the 439-year picontrol fit. Let *Q* denote discharge and *T*_*X*_(*Q*) be the function that yields the return period that corresponds to the discharge *Q* in model combination *X*. Its inverse $T_{X}^{-1}(T)$ then yields the discharge in model combination *X* corresponding to return period *T*. Thus, we used the following total return period function (see fig. S14 for an overview of the whole mapping procedure)

$$T_{\text{GCM},\text{GHM},r}(Q)\equiv T_{\text{GFDL}‐\text{ESM}2M,\text{LPJmL},\text{picontrol}}(T_{\text{GFDL}‐\text{ESM}2M,\text{LPJmL},r}^{-1}(T_{\text{GCM},\text{GHM},\text{hist}}(Q)))$$

Note that this is not necessarily a GEV distribution, but a distorted one where realization and picontrol run deviate. We assume that there is neither a trend in the picontrol run nor an autocorrelation between the different realizations. Because the historic period already includes a climate signal, it might deviate from the realizations; however, this trend is preserved by the mapping used, which only accounts for the differences between the realizations. In addition, the trend inside one of the periods is assumed to be significantly smaller than the difference between the historic and future period used.

For model bias correction, we followed the approach by Hirabayashi *et al*. (*5*): We mapped the return period from the procedure above to the corresponding flood depth in a MATSIRO (*21*) model run driven by observed climate forcing (*35*), in bins of 1-year (1 to 100) and 10-year (100 to 1000) return periods. Results from this observation-driven MATSIRO output have been shown to have realistic consistency in comparison with observation-based data. Details for this validation work are described in Hirabayashi *et al*. (*5*).

### Affected population

We then downscaled the flood depth using topological flood bed data of CaMa-Flood (to 0.005° grid and then reaggregated to 2.5′) to yield inundation area fraction on a 2.5′ grid. On the same grid resolution, we used aggregated 2010 gridded population data (Population Count, v4 2010) (*13*). For each cell and return period bin, inundation area multiplied by cell population yielded affected people. To save computational effort (and because each model run’s return period data are on a 0.25° resolution), we scaled up those to a 0.25° grid to obtain a mapping for return period to the affected population.

We expect our results to be optimistic regarding population distribution. Because we do not take into account scenarios of population distributions—but stick to the 2010 population distribution—for many of these regions, we expect an even higher number of affected population, particularly Africa and Southeast Asia. In addition, regions in coastal river deltas are under additional pressure of sea level rise through increased exposure to coastal floods.

### Region data

For cell-to-region mapping, we rasterized geo data from www.naturalearthdata.com to a 0.25° grid (10m-admin-0-countries_lakes, version 3.1.0, for the national level, and ne_10m_admin_1_states_provinces, version 3.0.0, for the subnational level) (*36*). To ensure accuracy and inclusion of small regions, we additionally used a 0.05° grid to correct the coarse grid according to the region with most cells in the corresponding finer grid cells. In addition, we advanced coastal cells toward the sea to capture population on coasts.

### Protection

The current flood protection at the subnational scale has recently been compiled in a global database, representing currently best global-scale knowledge in the maximum return period of flood that each country/region can prevent (*6*). Here, we used the Merged layer of the FLOPROS database (*6*), which combines empirical data about existing protection infrastructure (“Design layer”), data about protection standards and requirements set by policy measures (“Policy layer”), and model output from an observed relationship between gross domestic product per capita and flood protection (“Model layer”). These data were gridded to a 0.25° resolution with an expansion and raster correction as for the region data.

The large differences in increased high-end risk resulted not only from different changes in exposure to high-end floods but also from huge differences in the current protection, representing the vulnerability of regions; Africa sticks out in this regard. This has to be taken into account when interpreting our results with regard to changes in high-end flood levels but does not affect our results with regard to the question of changing risk. Accordingly, some of the differences between countries concerning required adaptation levels simply result from huge differences in current protection.

### High-end flood risk

Using the mapping for return period to affected population per cell, we created, for each model run, histograms of annual maximum flood events for each of the two periods (historical period, 1971 to 2004; future period, 2035 to 2044). There, only affected people for a return period larger than the cell’s current protection were counted. These were then aggregated to subnational regions, yielding a histogram per region per period and its 90th percentile for the high-end flood risk. In that, the histograms of all model runs were combined into one. For model combinations with only two RCP runs, these were counted twice in comparison to combinations with all four RCP runs available to ensure that equal weight was put on all hydrological/climate model combinations in the overall ensemble statistics.

This 90th percentile with 50 different model runs can be dominated by only a few hydrological/climate model combinations; the statistics is obtained over the affected population for each period (1700 data points for the historic period and 1680 data points for the future period; both are dominated by events not exceeding protection). These points are not necessarily independent; thus, the real sample size is probably smaller. However, this is already an improvement over previous work based on only one impact model, and we used several RCP scenarios to increase the ensemble size. An overview of how many of the climate models and hydrological models dominate the 90th percentile is given in figs. S7 and S8 and figs. S9 and S10, respectively. Two examples of a good and a bad model spread in that statistic are given in figs. S11 and S12. Although different climate models show particularly different trends in the high-end flood hazards for some regions, all climate models were justified using various assumptions and thus represent a possible projection of the future hazards. Given that we look at possible high-end risks, we should include those outcomes nevertheless in our statistics.

Other studies see further increase of flood frequency in the second half of the 21st century—for many world regions and for every carbon emission scenario (*5*). Consequently, we also expect high-end flood risk to further increase in those decades, especially if carbon emissions are not drastically reduced and more optimistic emission pathways are not taken.

### Necessary adaptation

For the future period, we repeated the previous procedure while consecutively increasing the return period protection by 1 year at each iteration in each cell of a subnational region. This procedure was continued until the 90th percentile of affected people equaled or fell below the value of the 90th percentile under the protection in the historic period. Then, we compared the affected population and the protection levels (see the next section) that the original and the increased protection fall into. This increment yielded the necessary adaptation needed to hold the historic high-end risk. The results shown are the median over all 12 realizations in the preindustrial control run as well as the 16.7 and 83.3 percentile as uncertainty ranges.

### Protection levels (return period bins)

We classified the current protection as well as the one necessary for keeping the high-end risk constant into levels according to their associated theoretical uncertainty. The adaptation pressure is then given as the number of protection levels necessary to bridge for constant high-end risk. The main source of statistical uncertainty is the insufficient number of discharge observation for a proper distribution fit. The cumulative distribution function of the GEV distribution with parameters μ, σ > 0, ξ for discharge *Q* is given as

$$F(Q)=\{\begin{array}{cc} \text{exp}(-\text{exp}(-\frac{Q-\mu }{\sigma })) & \text{for}\;\xi =0\\ \text{exp}(-{\left(1+\frac{Q-\mu }{\sigma }\xi \right)}^{-1/\xi }) & \text{for}\;\xi \neq 0 \end{array}$$

The return period associated with a discharge *Q* is the reciprocal of the probability of exceeding that value, *p* = 1 − *F*(*Q*),

$$T(Q)=\frac{1}{1-F(Q)}$$

Then, the error of a return period *T* with regard to the error in the distribution parameter μ is

$$\begin{array}{c} |\frac{\partial T}{\partial \mu }|=|\frac{F\;\text{ln}(F)}{\sigma }T^{2}{\left(\text{ln}(-F)\right)}^{\xi }|=\\ |\frac{1}{\sigma }(\text{ln}(T-1)-\;\text{ln}(T))(T-1)T{\left(\text{ln}(T)-\;\text{ln}(T-1)\right)}^{\xi }|\approx \\ \frac{T}{\sigma }{\left(\text{ln}(T)-\;\text{ln}(T-1)\right)}^{\xi }\;\text{for}\;T\gg 1 \end{array}$$

For ξ close to 0, this is proportional to *T* (analogously for σ and ξ), which is the case for most nondry regions; otherwise, ξ goes with *T*^2^. Accordingly, we chose protection levels with widths proportional to the corresponding return period, that is, exponentially increasing level bounds. To have a sufficient number of levels, we simply stuck to powers of 2; thus, our level bounds were 0, 1, 2, 4, 8, 16, 32, 64, 128, 256, 512, and 1000; the lower bound was included, whereas the upper one was excluded. These assumptions about the levels are only precise for the Gumbel distribution (ξ = 0); nevertheless, our results further hold qualitatively when restricting to that distribution (see fig. S17).

## Supplementary Material

http://advances.sciencemag.org/cgi/content/full/4/1/eaao1914/DC1

## SUPPLEMENTARY MATERIALS

Supplementary material for this article is available at http://advances.sciencemag.org/cgi/content/full/4/1/eaao1914/DC1 fig. S1. Increase in the regional flood protection level required to preserve the current high-end flood risk for the period 2035 to 2044 (realization 16.7 percentile, lower likely range). fig. S2. Increase in the regional flood protection level required to preserve the current high-end flood risk for the period 2035 to 2044 (realization 83.3 percentile, upper likely range). fig. S3. Required adaptation relative to current protection to preserve the current high-end flood risk for the period 2035 to 2044 (realization ensemble median). fig. S4. Affected people in the historic period. fig. S5. Affected people in the future period. fig. S6. Absolute increase in high-end flood risk. fig. S7. Climate model agreement (historic period). fig. S8. Climate model agreement (future period). fig. S9. Hydrological model agreement (historic period). fig. S10. Hydrological model agreement (future period). fig. S11. Example histogram of affected people (in India). fig. S12. Example histogram of affected people (in Egypt). fig. S13. Zoomed-in views of selected metropolitan areas; increase in the regional flood protection level required to preserve the current high-end flood risk for the period 2035 to 2044. fig. S14. Schematic of the method to yield the affected population from discharge. fig. S15. Probability plot correlation coefficient for the preindustrial control run of 439 years as a goodness of (GEV) fit measure. fig. S16. Probability density functions for the fitted GEV distribution at four representative grid cells (hot/cold and wet/dry). fig. S17. Increase in the regional flood protection level required to preserve the current high-end flood risk for the period 2035 to 2044 (realization ensemble median) using the Gumbel distribution for the extreme value fit (cf. Fig. 3 for GEV fit). table S1. Main characteristics of the GHMs as used in this study, based on the study of Warszawski *et al*. (*7*). CSV (comma-separated-values) file of the raw data
