# Supplementary material for: Adaptation required to preserve future high-end river flood risk at present levels
Source: Sci Adv. 2018 Jan 10;4(1):eaao1914. doi: 10.1126/sciadv.aao1914 (PMC5762193; doi:10.1126/sciadv.aao1914)
Supplement: http://advances.sciencemag.org/cgi/content/full/4/1/eaao1914/DC1 [file supp_4_1_eaao1914__index.html]

Science Advances | Science Advances

## Supplementary Materials

**This PDF file includes:**

- fig. S1. Increase in the regional flood protection level required to preserve the current high-end flood risk for the period 2035 to 2044 (realization 16.7 percentile, lower likely range).
- fig. S2. Increase in the regional flood protection level required to preserve the current high-end flood risk for the period 2035 to 2044 (realization 83.3 percentile, upper likely range).
- fig. S3. Required adaptation relative to current protection to preserve the current high-end flood risk for the period 2035 to 2044 (realization ensemble median).
- fig. S4. Affected people in the historic period.
- fig. S5. Affected people in the future period.
- fig. S6. Absolute increase in high-end flood risk.
- fig. S7. Climate model agreement (historic period).
- fig. S8. Climate model agreement (future period).
- fig. S9. Hydrological model agreement (historic period).
- fig. S10. Hydrological model agreement (future period).
- fig. S11. Example histogram of affected people (in India).
- fig. S12. Example histogram of affected people (in Egypt).
- fig. S13. Zoomed-in views of selected metropolitan areas; increase in the regional flood protection level required to preserve the current high-end flood risk for the period 2035 to 2044.
- fig. S14. Schematic of the method to yield the affected population from discharge.
- fig. S15. Probability plot correlation coefficient for the preindustrial control run of 439 years as a goodness of (GEV) fit measure.
- fig. S16. Probability density functions for the fitted GEV distribution at four representative grid cells (hot/cold and wet/dry).
- fig. S17. Increase in the regional flood protection level required to preserve the current high-end flood risk for the period 2035 to 2044 (realization ensemble median) using the Gumbel distribution for the extreme value fit (cf. Fig. 3 for GEV fit).
- table S1. Main characteristics of the GHMs as used in this study, based on the study of Warszawski et al. (*7*).

Download PDF

**Other Supplementary Material for this manuscript includes the following:**

- CSV (comma-separated-values) file of the raw data (Microsoft Excel format)

**Files in this Data Supplement:**

- Adobe PDF - aao1914\_SM.pdf
