# Supplementary material for: Adaptation required to preserve future high-end river flood risk at present levels
Source: Sci Adv. 2018 Jan 10;4(1):eaao1914. doi: 10.1126/sciadv.aao1914 (PMC5762193; doi:10.1126/sciadv.aao1914)
Supplement: http://advances.sciencemag.org/cgi/content/full/4/1/eaao1914/DC1 [file aao1914_SM.pdf]

Science Advances

AAAS

advances.sciencemag.org/cgi/content/full/4/1/eaao1914/DC1

Supplementary Materials for

Adaptation required to preserve future high-end river flood risk at present levels

Sven N. Willner, Anders Levermann, Fang Zhao, Katja Frieler

Published 10 January 2018, *Sci. Adv.* **4**, eaao1914 (2018)
DOI: 10.1126/sciadv.aao1914

The PDF file includes:

- fig. S1. Increase in the regional flood protection level required to preserve the current high-end flood risk for the period 2035 to 2044 (realization 16.7 percentile, lower likely range).
- fig. S2. Increase in the regional flood protection level required to preserve the current high-end flood risk for the period 2035 to 2044 (realization 83.3 percentile, upper likely range).
- fig. S3. Required adaptation relative to current protection to preserve the current high-end flood risk for the period 2035 to 2044 (realization ensemble median).
- fig. S4. Affected people in the historic period.
- fig. S5. Affected people in the future period.
- fig. S6. Absolute increase in high-end flood risk.
- fig. S7. Climate model agreement (historic period).
- fig. S8. Climate model agreement (future period).
- fig. S9. Hydrological model agreement (historic period).
- fig. S10. Hydrological model agreement (future period).
- fig. S11. Example histogram of affected people (in India).
- fig. S12. Example histogram of affected people (in Egypt).
- fig. S13. Zoomed-in views of selected metropolitan areas; increase in the regional flood protection level required to preserve the current high-end flood risk for the period 2035 to 2044.
- fig. S14. Schematic of the method to yield the affected population from discharge.
- fig. S15. Probability plot correlation coefficient for the preindustrial control run of 439 years as a goodness of (GEV) fit measure.
- fig. S16. Probability density functions for the fitted GEV distribution at four representative grid cells (hot/cold and wet/dry).

- fig. S17. Increase in the regional flood protection level required to preserve the current high-end flood risk for the period 2035 to 2044 (realization ensemble median) using the Gumbel distribution for the extreme value fit (cf. Fig. 3 for GEV fit).
- table S1. Main characteristics of the GHMs as used in this study, based on the study of Warszawski *et al*. (*7*).

Other Supplementary Material for this manuscript includes the following:
(available at advances.sciencemag.org/cgi/content/full/4/1/eaao1914/DC1)

- CSV (comma-separated-values) file of the raw data (Microsoft Excel format)

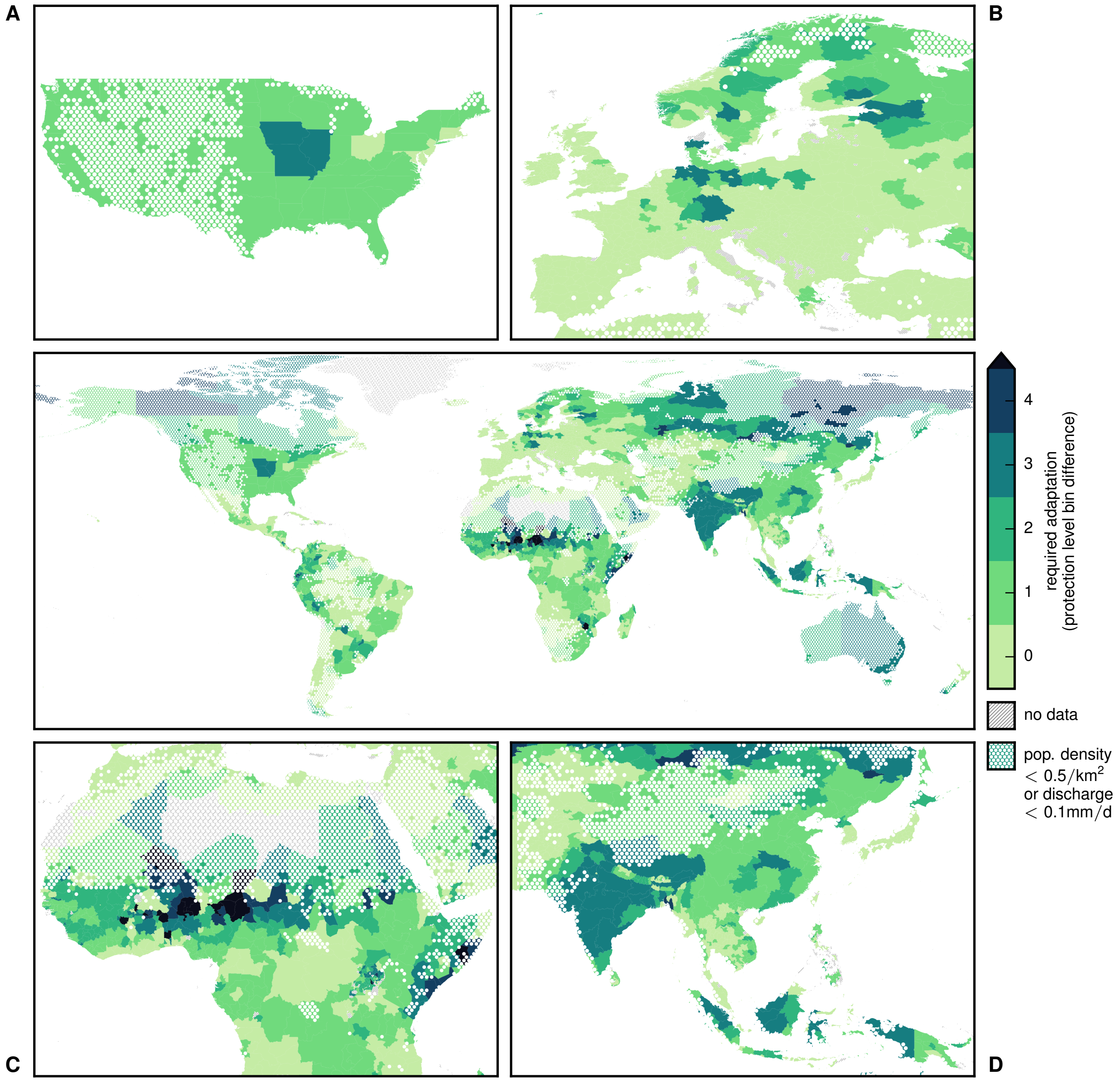

fig. S1. Increase in the regional flood protection level required to preserve the current high-end flood risk for the period 2035 to 2044 (realization 16.7 percentile, lower likely range). Additional protection is given in levels, starting with 0 for regions without adaptation need. Level boundaries are 0, 1, 2, 4, 8, 16, 32, 64, 128, 256, 512, and 1000. Numbers shown are absolute difference in level numbers to current protection per sub-national region in the FLOPROS database (*6*). Sub-figures show regional foci on the USA (a), Europe (b), Africa (c), and Southeast and East Asia (d).

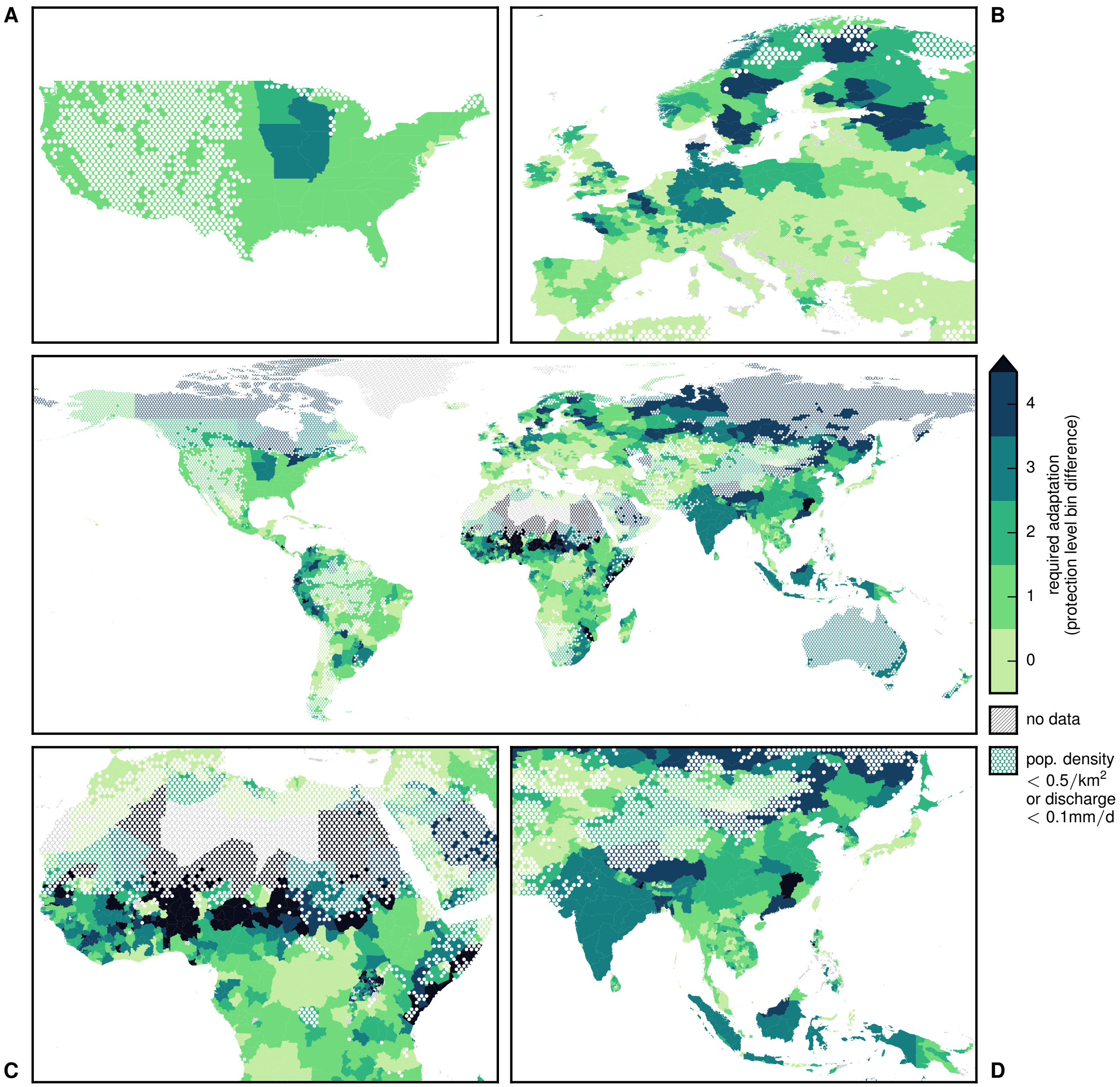

fig. S2. Increase in the regional flood protection level required to preserve the current high-end flood risk for the period 2035 to 2044 (realization 83.3 percentile, upper likely range). Additional protection is given in levels, starting with 0 for regions without adaptation need. Level boundaries are 0, 1, 2, 4, 8, 16, 32, 64, 128, 256, 512, and 1000. Numbers shown are absolute difference in level numbers to current protection. Sub-figures show regional foci on the USA (a), Europe (b), Africa (c), and Southeast and East Asia (d).

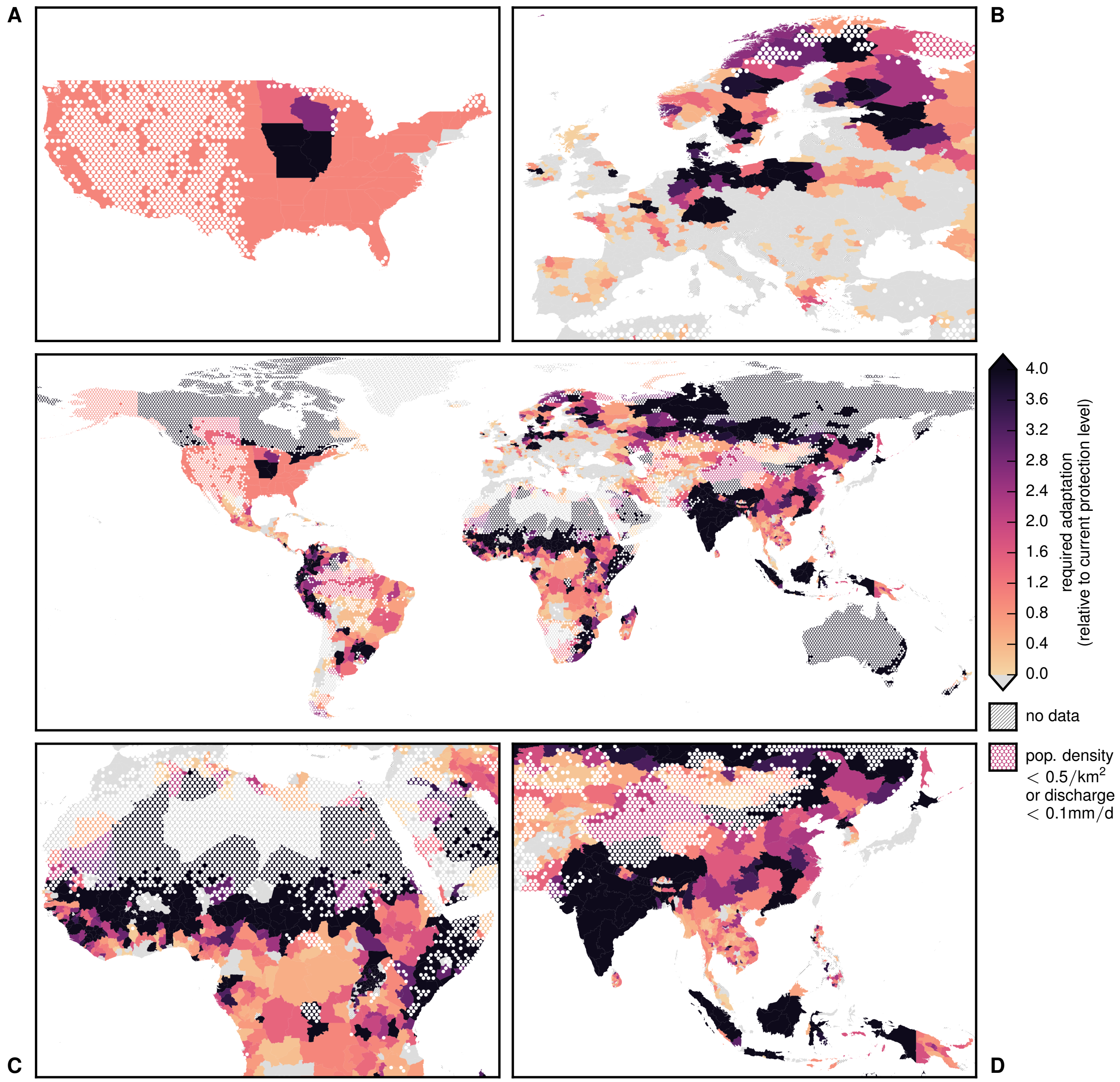

fig. S3. Required adaptation relative to current protection to preserve the current high-end flood risk for the period 2035 to 2044 (realization ensemble median). Numbers shown are relative difference (in multiples of current protection level) in return period in years protected against. Sub-figures show regional foci on the USA (a), Europe (b), Africa (c), and Southeast and East Asia (d).

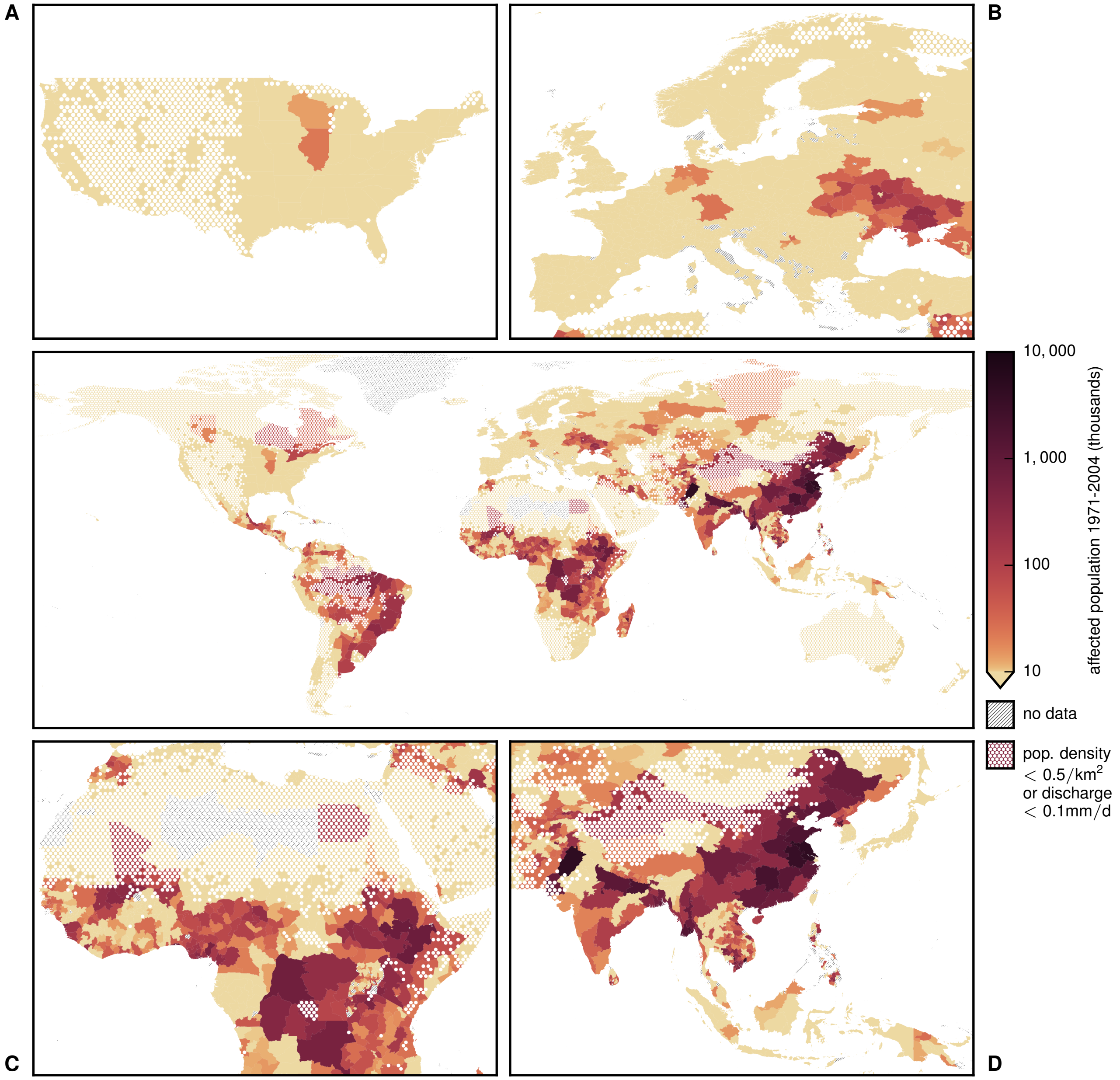

fig. S4. Affected people in the historic period. High-end flood risk (90th percentile) per sub-national region given in number of affected people (logarithmic plot); historic period 1971-2004.

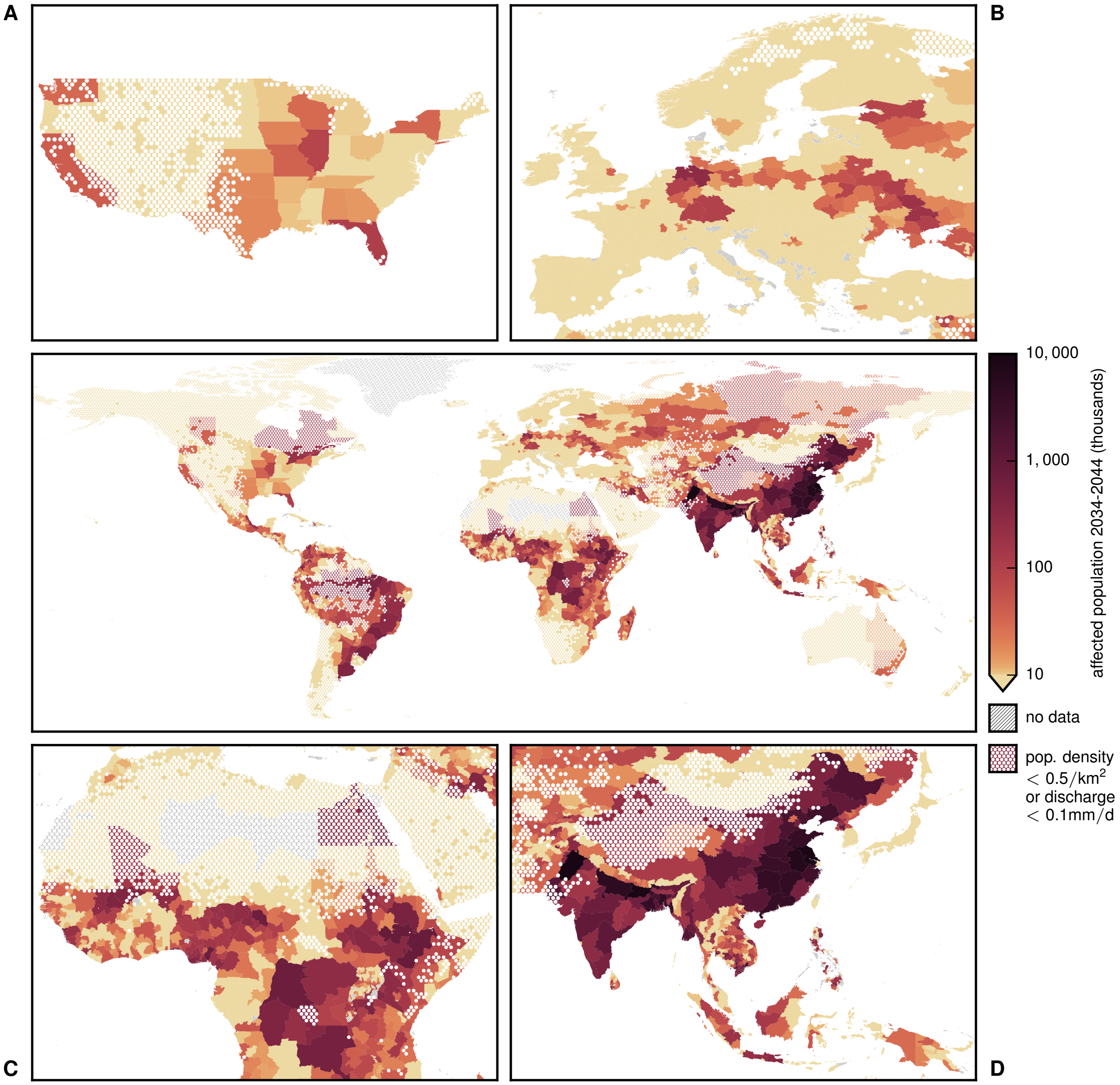

fig. S5. Affected people in the future period. High-end flood risk (90th percentile) per sub-national region given in number of affected people (logarithmic plot); future period 2035-2044.

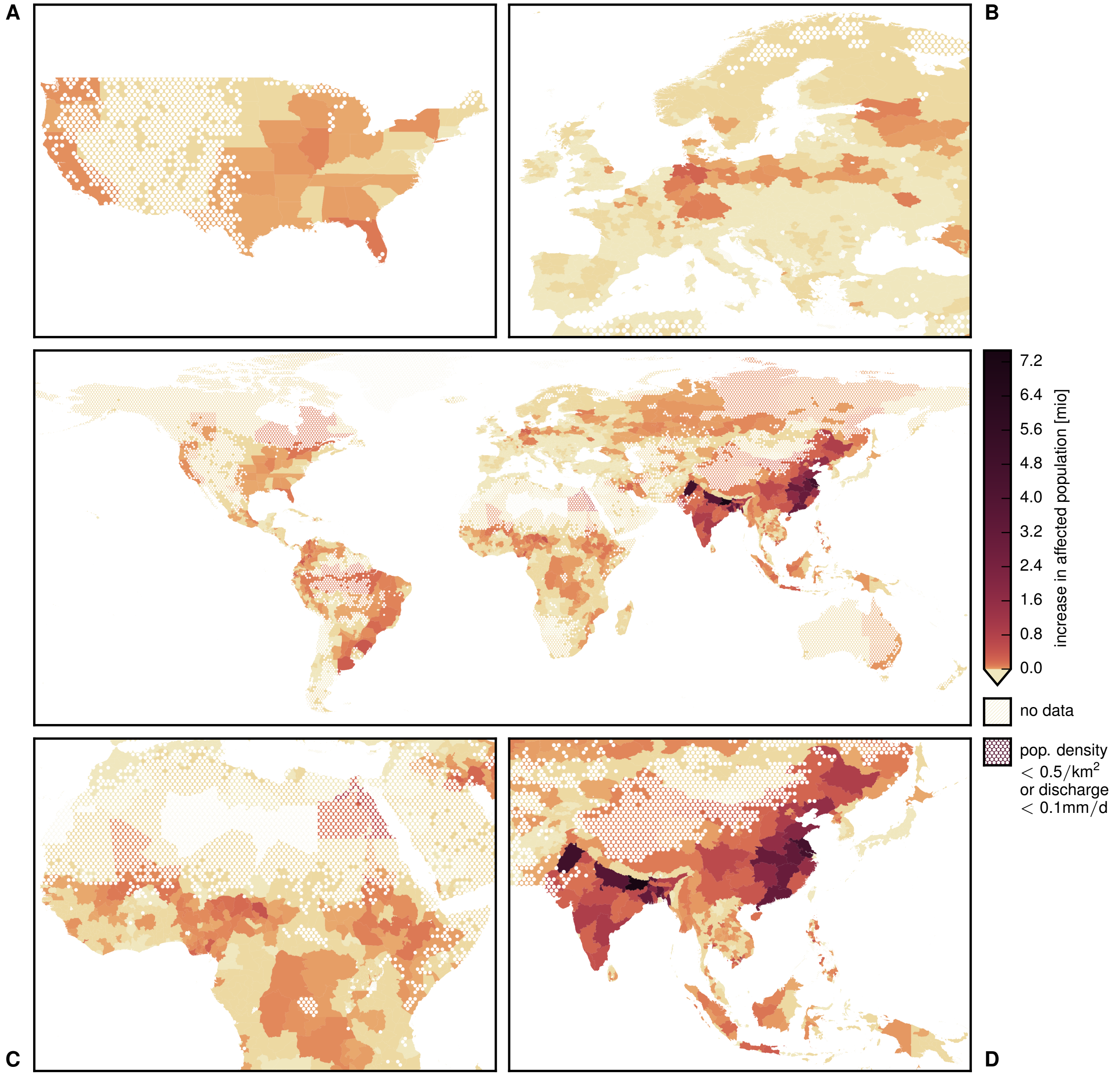

fig. S6. Absolute increase in high-end flood risk. Difference in high-end flood risk (90th percentile) per sub-national region given in absolute number of additionally affected people (lower bound 0); historic period 1971-2004, future period 2035-2044.

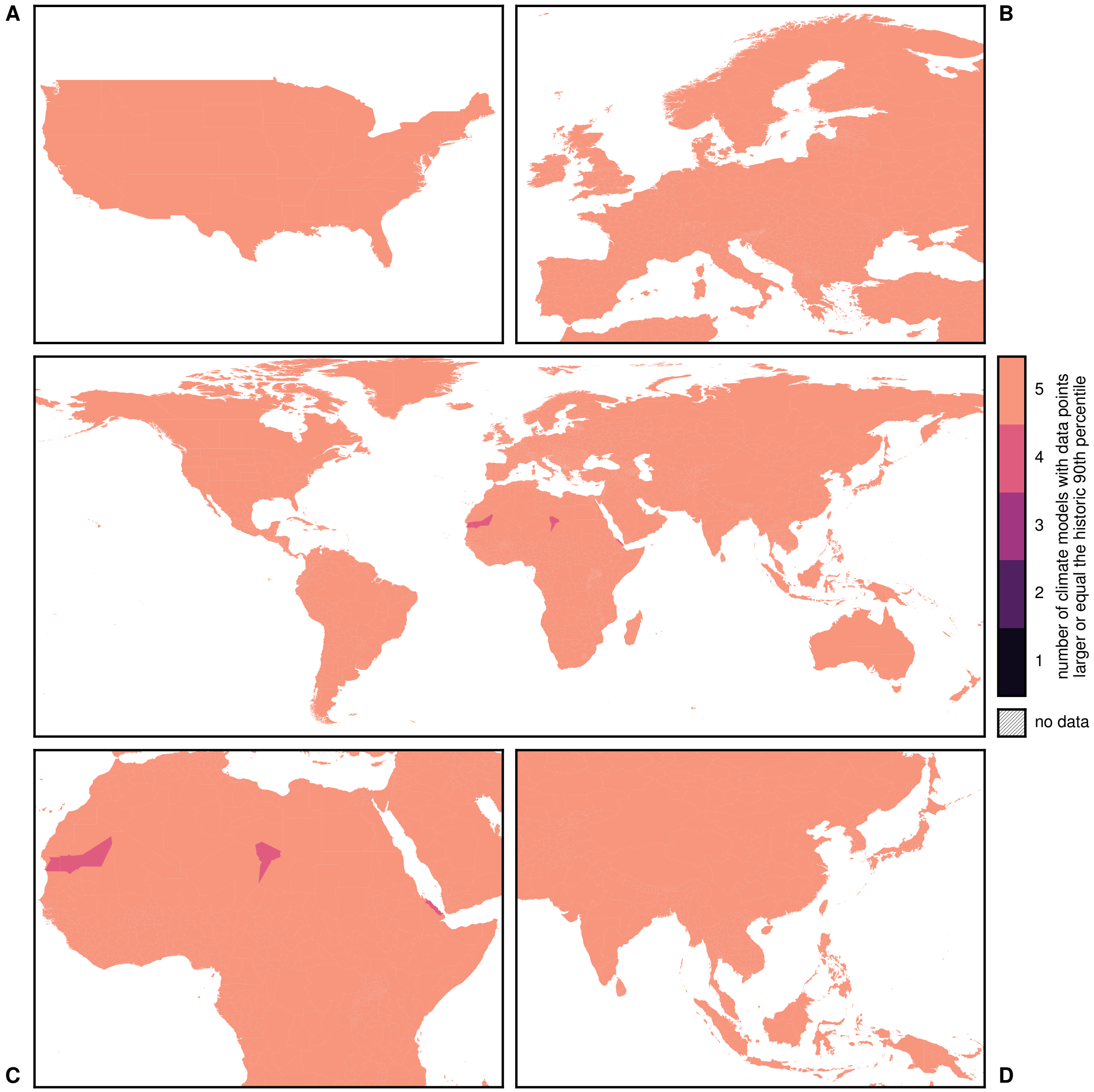

fig. S7. Climate model agreement (historic period). Number of climate models with data points (i. e. annual maximum flood events) larger or equal the historic overall 90th percentile (without correcting for realizations of pre-industrial control run); over all ten hydrological models per climate model run.

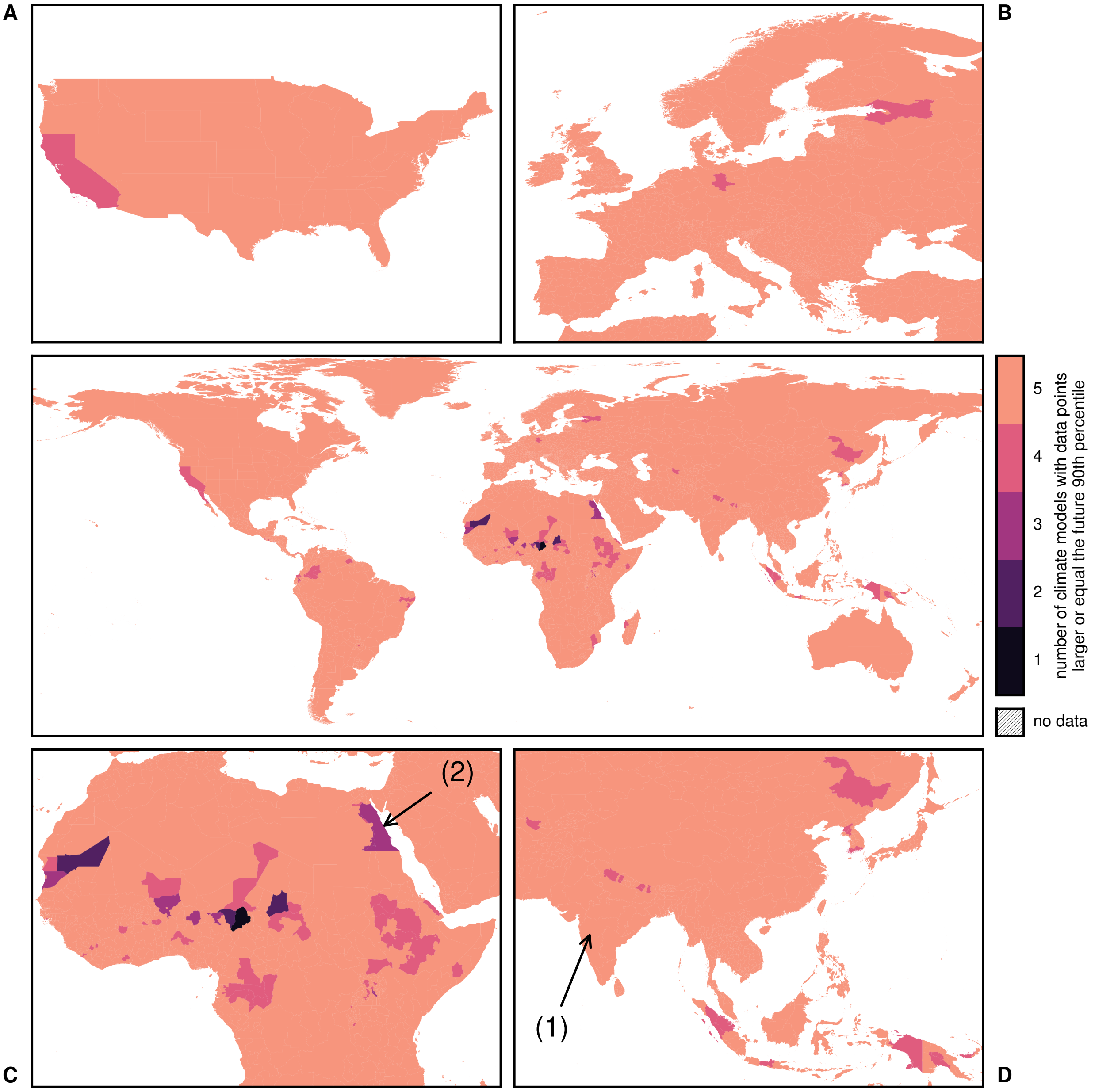

fig. S8. Climate model agreement (future period). Number of climate models with data points (i. e. annual maximum flood events) larger or equal the future overall 90th percentile (without correcting for realizations of pre-industrial control run); over all ten hydrological models per climate model run. The regions of histogram in figs. S11 and S12 are marked by (1) and (2), respectively.

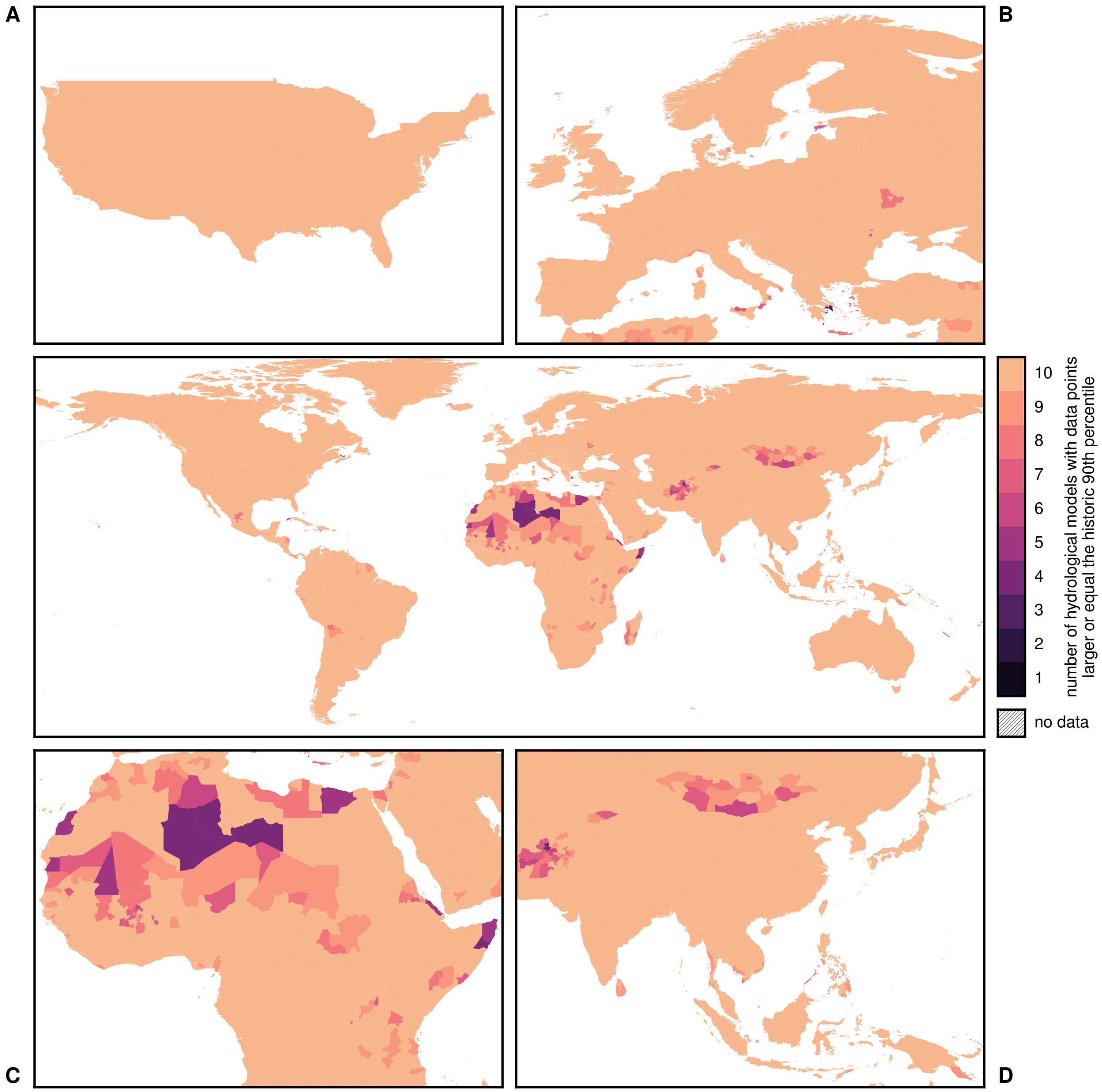

fig. S9. Hydrological model agreement (historic period). Number of hydrological models with data points (i. e. annual maximum flood events) larger or equal the historic overall 90th percentile (without correcting for realizations of pre-industrial control run); over all five climate models per hydrological model run.

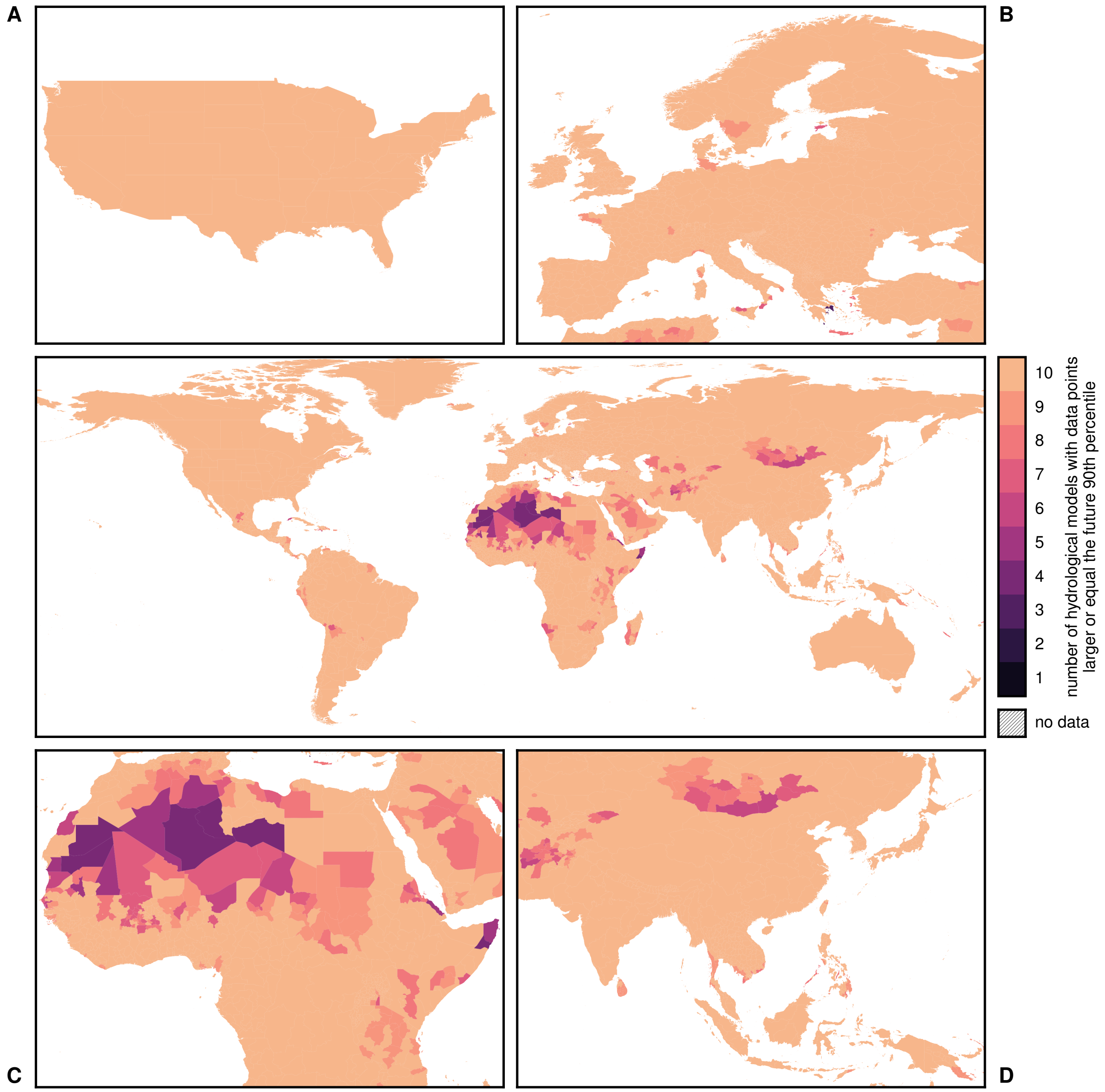

fig. S10. Hydrological model agreement (future period). Number of hydrological models with data points (i. e. annual maximum flood events) larger or equal the future overall 90th percentile (without correcting for realizations of pre-industrial control run); over all five climate models per hydrological model run.

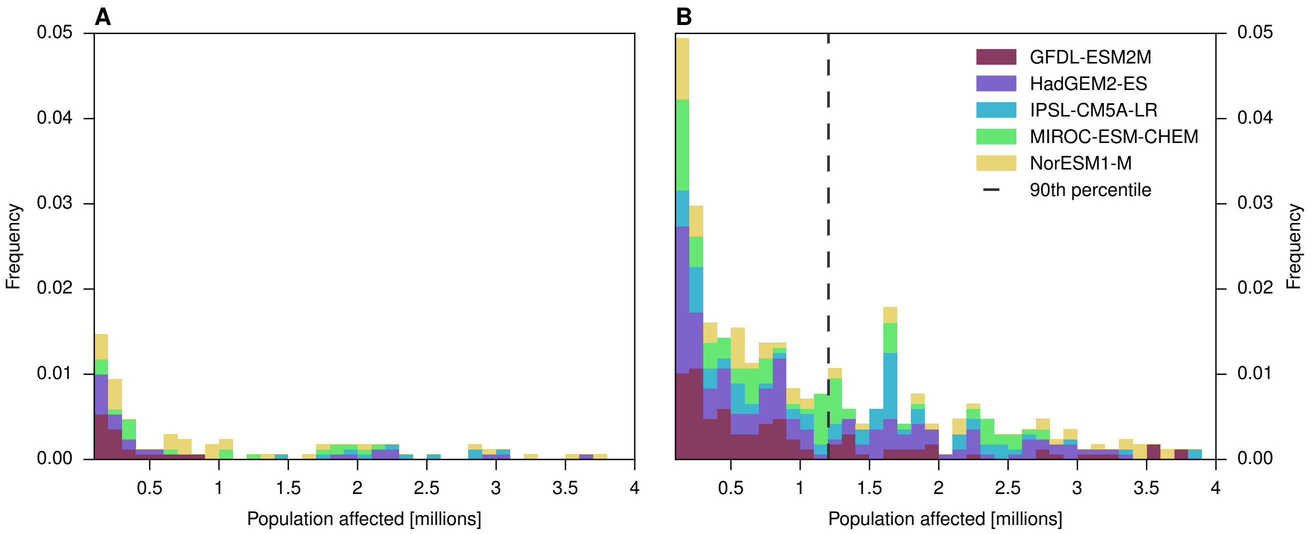

fig. S11. Example histogram of affected people (in India). Histogram of affected people (annual maximum of daily affected people) for the historic period (**a**) and the future period (**b**). Example for Maharashtra state in India as marked (1) in fig. 8. Model years are binned into 0.1 million per bin (first bin excluded due to high frequency) and are differentiated by color for different climate models. All climate models contribute to the 90th percentile of the overall distribution (without correcting for realizations of pre-industrial control run).

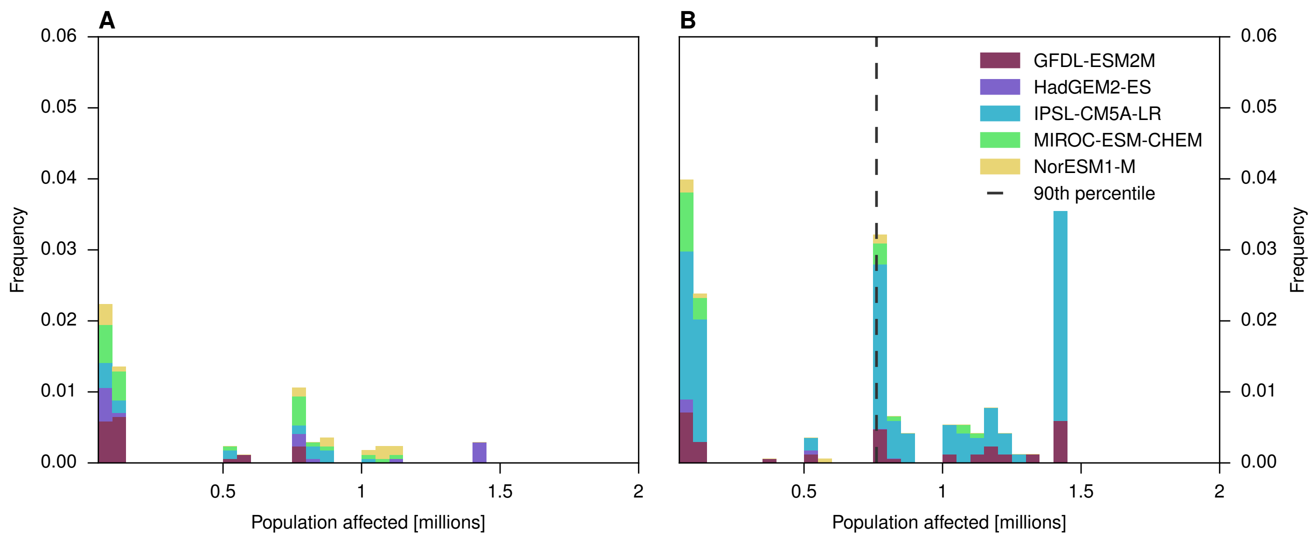

fig. S12. Example histogram of affected people (in Egypt). Histogram of affected people (annual maximum of daily affected people) for the historic period (**a**) and the future period (**b**). Example for the Red Sea Governate (Al Bahr al Ahmar) in Egypt as marked (2) in fig. S8. Model years are binned into 0.05 million per bin (first bin excluded due to high frequency) and are differentiated by color for different climate models. Only three climate models contribute to the 90th percentile of the overall distribution for the future period (without correcting for realizations of pre-industrial control run).

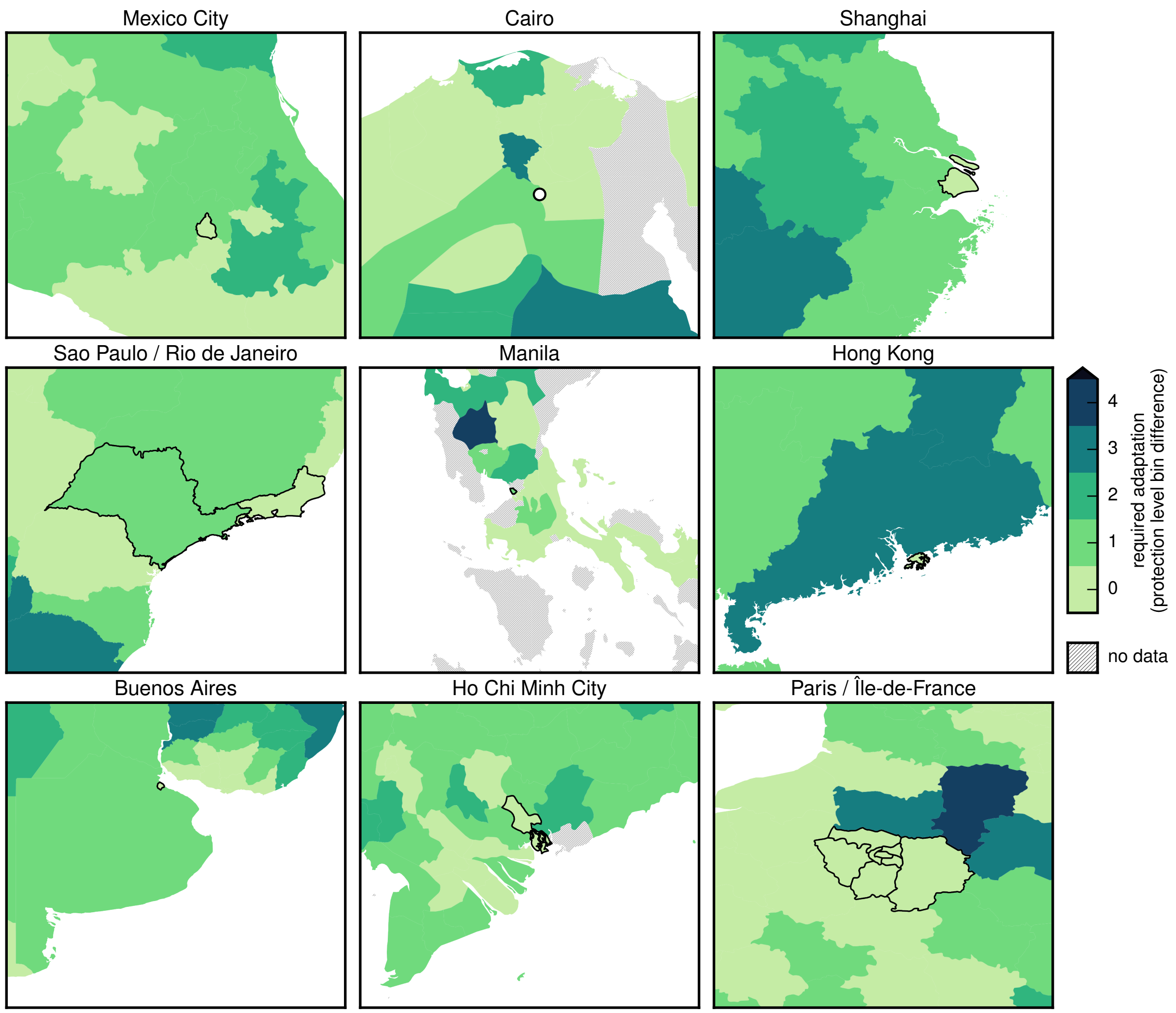

fig. S13. Zoomed-in views of selected metropolitan areas; increase in the regional flood protection level required to preserve the current high-end flood risk for the period 2035 to 2044. Numbers and sub-national areas as in Fig. 2. For each sub-figure the cities mentioned (or corresponding sub-regions) in the title are indicated by black outlines. Metropolitan regions chosen here show a lower adaptation need than their surrounding areas, mostly due to already higher protection. Though some are coastal areas, numbers are only for fluvial flood risk.

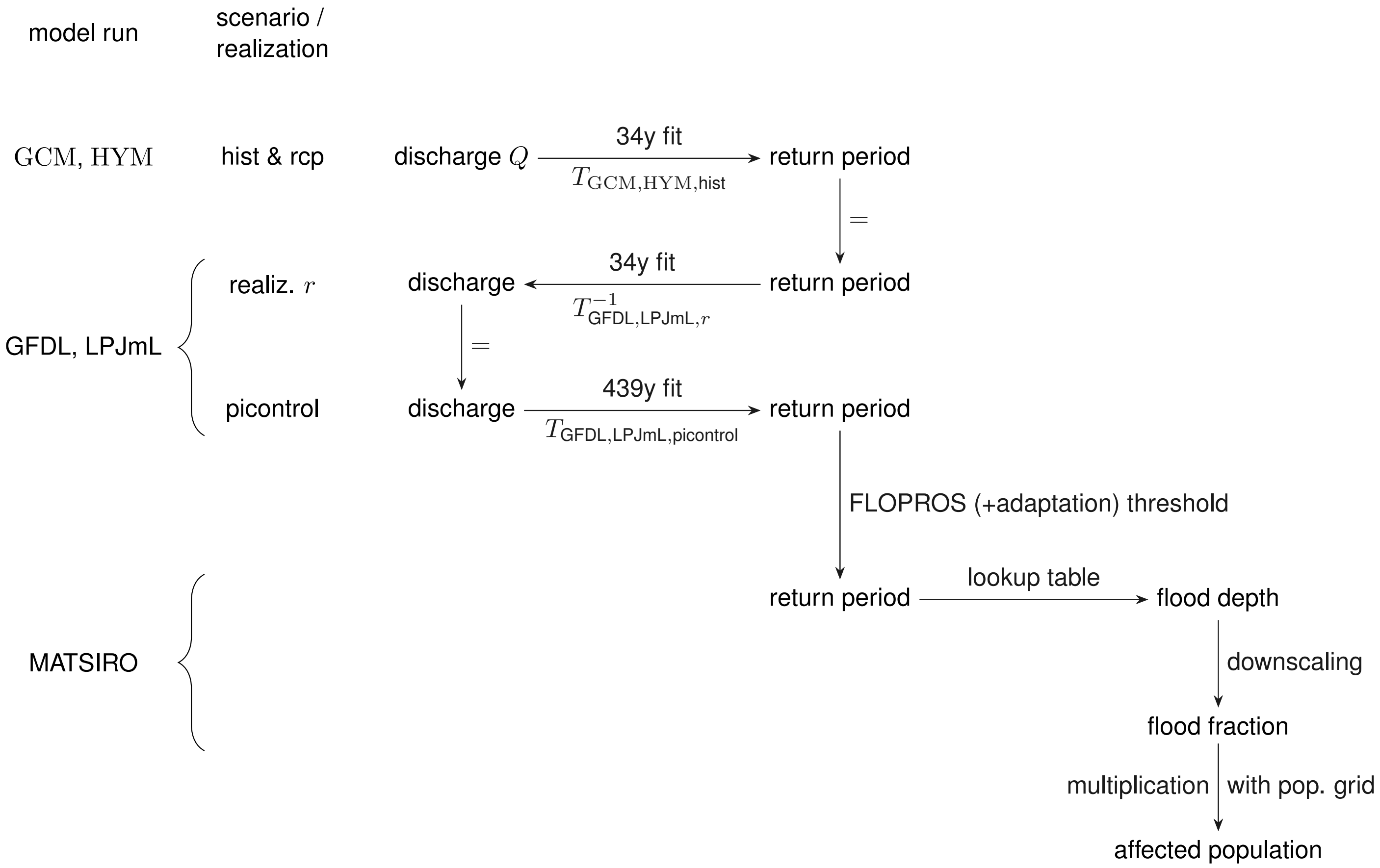

fig. S14. Schematic of the method to yield the affected population from discharge. It includes bias correction for model run biases as well as for bias because of only 34 years used for extreme value fit.

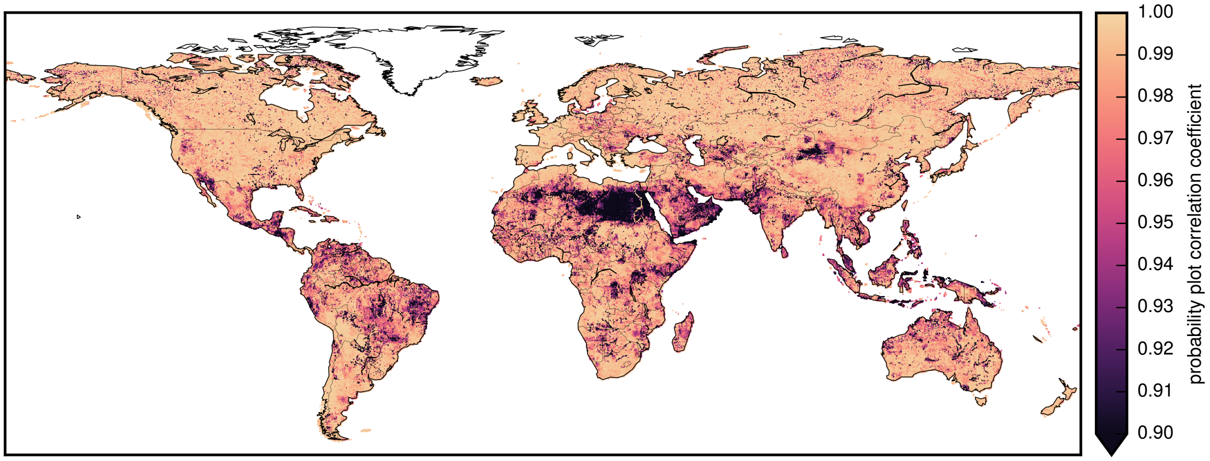

fig. S15. Probability plot correlation coefficient for the preindustrial control run of 439 years as a goodness of (GEV) fit measure. The Generalized Extreme Value distribution very well describes the observed annual maximum daily discharge for almost all non-dry grid cells (cf. Figure 1).

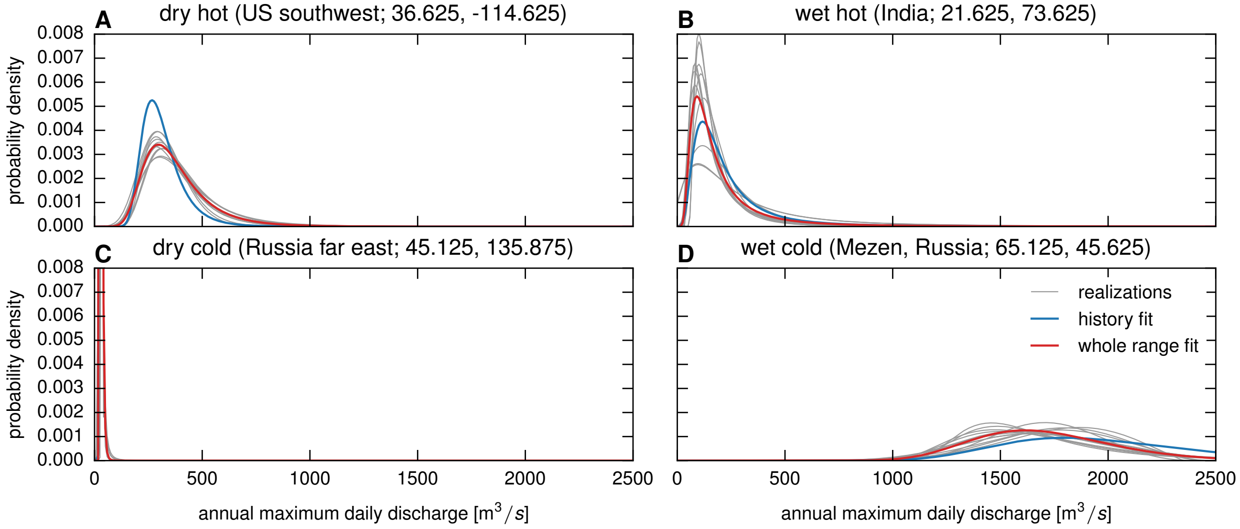

fig. S16. Probability density functions for the fitted GEV distribution at four representative grid cells (hot/cold and wet/dry). Shown are fits for the 12 realizations, one without correction for the pre-industrial control run ("history fit"), and the pre-industrial control run itself with 439 years ("whole range fit").

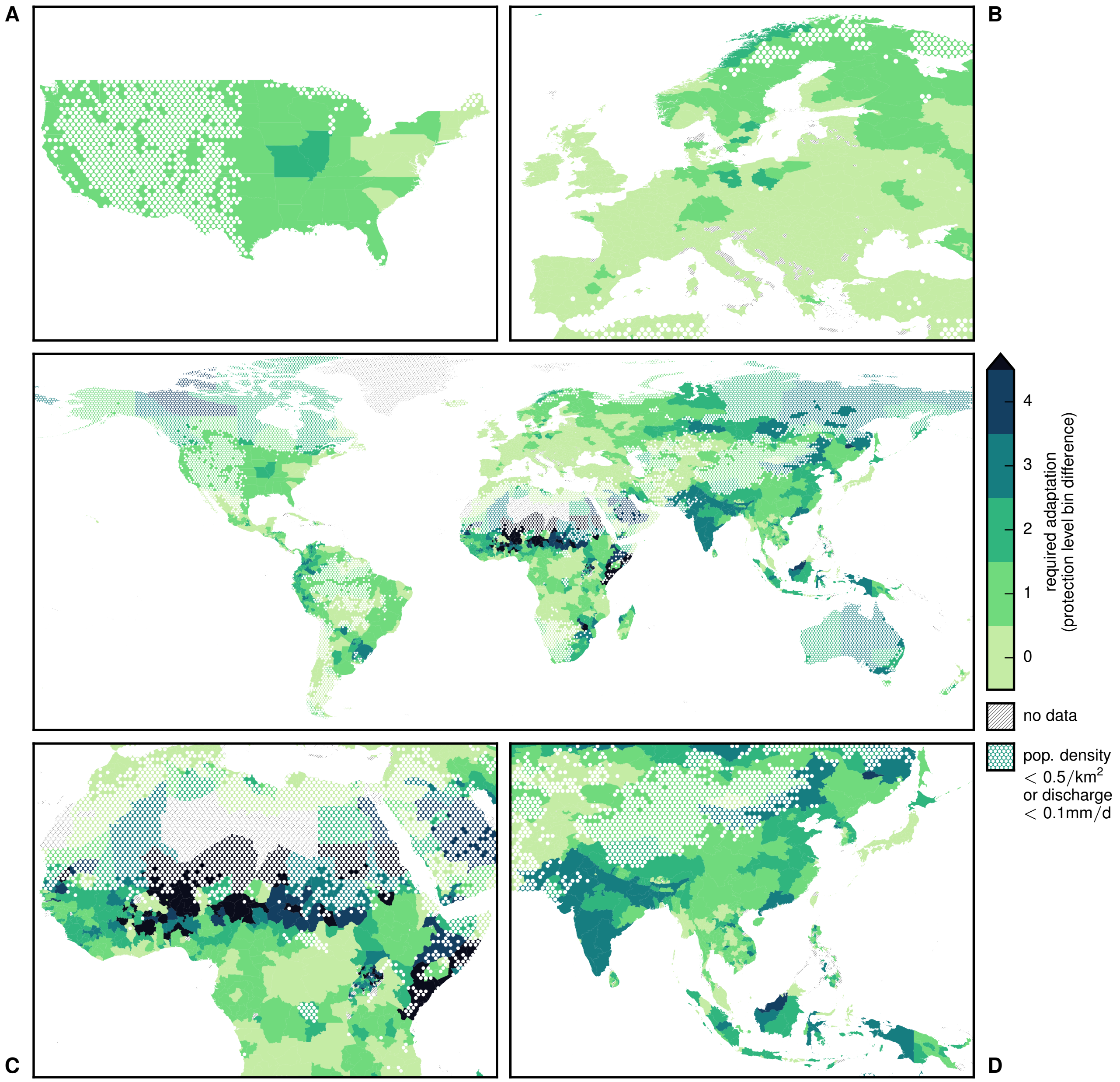

fig. S17. Increase in the regional flood protection level required to preserve the current high-end flood risk for the period 2035 to 2044 (realization ensemble median) using the Gumbel distribution for the extreme value fit (cf. Fig. 3 for GEV fit). Additional protection is given in levels, starting with 0 for regions without adaptation need. Level boundaries are 0, 1, 2, 4, 8, 16, 32, 64, 128, 256, 512, and 1000. Numbers shown are absolute difference in level numbers to current protection. Sub-figures show regional foci on the USA (a), Europe (b), Africa (c), and Southeast and East Asia (d).

table S1. Main characteristics of the GHMs as used in this study, based on the study of Warszawski *et al*. (*7*).

Model name	Time step length	Meteorological forcing[a]	Energy balance	Evaporation scheme[b]	Runoff scheme[c]	Snow scheme	Vegetation dynamics	CO_2 effect[d]	References
DBH	1h	P, T, W, Q, LW, SW, SP	Yes	Energy balance	Infiltration excess	Energy balance	No	Constant	(Tang et al. 2007b; Tang et al. 2008)
H08	Daily	R, S, T, W, Q, LW, SW, SP	Yes	Bulk formula	Saturation excess, non-linear	Energy balance	No	No	(Hanasaki et al. 2008a; Hanasaki et al. 2008b)
JULES	30 mins	R, S, T, W, Q, LW, SW, SP	Yes	Penman-Monteith	Infiltration excess, saturation excess, groundwater.	Energy balance	Yes	Varying	(Best et al. 2011; Clark et al. 2011)
LPJmL	Daily	P, T, LW_n, SW	No	Priestley-Taylor	Saturation excess	Degree-day	Yes	Varying	(Bondeau et al. 2007; Rost et al. 2008)
Mac-PDM.09	Daily	P, T, W, Q, LW_n, SW	No	Penman-Monteith	Saturation excess, non-linear	Degree-day	No	No	(Arnell 1999; Gosling and

									Arnell 2011)
MATSIRO	1 hr	R, S, T, W, Q, LW, SW, SP	Yes	Bulk formula	Infiltration excess, saturation excess, groundwater.	Energy balance	No	Constant	(Takata, Emori, and Watanabe 2003; Pokhrel et al. 2012)
MPI-HM	Daily	P, T, W, Q, LW_n, SW, SP	No	Penman-Monteith	Saturation excess, non-linear	Degree-day	No	No	(Hagemann and Dümenil Gates 2003; Stacke and Hagemann 2012)
PCR-GLOBWB	Daily	P,T	No	Hamon	Saturation Excess Beta Function	Degree Day	No	No	(Wada et al. 2010; van Beek, Wada, and Bierkens 2011; Wada et al. 2011)
VIC	Daily, 3hr snow	P, T, W, Q, LW, SW, SP.	Only for snow.	Penman-Monteith	Saturation excess, non-linear	Energy balance.	No	No	(Liang et al. 1994; Lohmann and Raschke

									1998)
WBM	Daily	P,T	No	Hamon	Saturation Excess	Empirical temp and precip based formula	No	No	(Vörösmarty, Federer, and Schloss 1998; Wisser et al. 2010)

[a] R: rainfall rate, S: snowfall rate, P: precipitation rate (rain and snow calculated in the model), T: air temperature, W: wind speed, Q: air specific humidity, LW: downwelling longwave radiation; LW_n: net longwave radiation; SW: downwelling shortwave radiation, SP: surface pressure.

[b] Bulk formula: Bulk transfer coefficients are used when calculating turbulent heat fluxes.

[c] Non-linear: Subsurface runoff is a non-linear function of soil moisture.

[d] CO_2 concentration in calculation of stomatal conductance.
